# Breaking Barriers and Fostering Inclusion: The Success of the Culturally Attuned and Responsive Engagement (CARE) Model among Black Americans, Hispanics/Latinos, and Africans in Aging and Alzheimer’s Disease Research

**DOI:** 10.1002/alz.093443

**Published:** 2025-01-09

**Authors:** Rhoda Moise, Dingtian Cai, Kara L. Hamilton‐Nelson, Larry D. Adams, Farid Rajabli, Michael L. Cuccaro, Brian W. Kunkle, Jeffery M. Vance, Christiane Reitz, Giuseppe Tosto, William S. Bush, Jonathan L. Haines, Goldie S. Byrd, Rufus O. Akinyemi, Adesola Ogunniyi, Margaret A Pericak‐Vance, Azizi A Seixas, Susan Halloran Blanton

**Affiliations:** ^1^ Department of Psychiatry and Behavioral Sciences, University of Miami Miller School of Medicine, Miami, FL USA; ^2^ John P. Hussman Institute for Human Genomics, University of Miami Miller School of Medicine, Miami, FL USA; ^3^ John P. Hussman Institute for Human Genomics, Miami, FL USA; ^4^ Dr. John T. Macdonald Foundation Department of Human Genetics, University of Miami Miller School of Medicine, Miami, FL USA; ^5^ Dr. John T. Macdonald Foundation Department of Human Genetics, Miller School of Medicine, University of Miami, Miami, FL USA; ^6^ Gertrude H. Sergievsky Center, Taub Institute for Research on the Aging Brain, Departments of Neurology, Psychiatry, and Epidemiology, College of Physicians and Surgeons, Columbia University, New York, NY USA; ^7^ Department of Population and Quantitative Health Sciences, Institute for Computational Biology, Case Western Reserve University, Cleveland, OH USA; ^8^ Maya Angelou Center for Health Equity, Wake Forest University School of Medicine, Winston‐Salem, NC USA; ^9^ Neuroscience and Aging Research Unit, Institute of Advanced Medical Research and Training, College of Medicine, University of Ibadan, Ibadan Nigeria; ^10^ Africa Dementia Consortium, Ibadan, Ibadan Nigeria; ^11^ College of Medicine, University of Ibadan, Ibadan, Oyo State Nigeria; ^12^ Department of Informatics and Health Science Data, University of Miami Miller School of Medicine, Miami, FL USA

## Abstract

**Background:**

Black Americans (BAs), Hispanics/Latinos (H/Ls), and Africans (As) face a disproportionate burden of aging and Alzheimer’s Disease and Related Dementias (AD/ADRD), coupled with underrepresentation in research. Further, researchers also report a lack of compliance on sensitive social determinants of health data for AD/ADRD research. For instance, the PRAPARE tool reports a low completion rate in community and clinical settings. Understanding/addressing the barriers and facilitators to research engagement in these communities is essential for enhancing our understanding of disparities and bolstering representation in aging research. Based on these findings, we developed and implemented a novel recruitment and engagement research framework, Culturally Attuned and Responsive Engagement (CARE) Model. This study aims to describe the success of CARE framework to foster inclusive research engagement, enhancing recruitment, retention, and outcomes among BAs, H/Ls, As.

**Method:**

Initially, we utilized semi‐structured interviews to elicit in‐depth discussions around cultural sensitivity of health research. Next, employing item response theory, we developed a comprehensive Social Determinants of Health (SDOH) instrument tailored for BAs, H/Ls, and As. We included a focus on multilevel determinants at individual, interpersonal, and institutional levels. For example, variables spanned education, employment, housing, finances, nutrition, social engagement and isolation, transportation, exposure to violence, discrimination, disability, health behaviors, resilience, sanitation, and religion. The success of the CARE Model was assessed through completion rates.

**Results:**

Domestic sites with BAs and H/Ls(N = 1022) demonstrated a 57% completion rate, with 586 participants completing SDOH data. International sites with As (N = 465) exhibited a similar completion rate of 57%, with 266 participants completing SDOH data. The CARE Model’s approach, emphasizing personalized outreach, decentralized engagement, and cultural competency, contributed to its success in achieving high completion rates. CARE demonstrates utility across different contexts for different underrepresented groups.

**Conclusion:**

The CARE Model represents a groundbreaking shift towards a more inclusive, responsive, and participant‐centered research landscape. By prioritizing cultural competency, trust‐building, and open dialogue, this innovative approach addresses historical barriers, enhancing representation and providing valuable insights into disparities within Black American and Hispanic/Latino populations. The CARE Model serves as a tool for future research endeavors seeking to bridge gaps and foster true inclusivity in aging and AD/ADRD studies.

